# Attitude and practices of tracheostomy care among nursing staff in Saudi Arabia

**DOI:** 10.1186/s12912-022-01150-3

**Published:** 2022-12-23

**Authors:** Turki M. Bin Mahfoz

**Affiliations:** Department of Otolaryngology, College of Medicine, Imam Mohammad Ibn Saud Islamic University (IMSIU), Riyadh, 13317 Saudi Arabia

**Keywords:** Tracheostomy, Confidence, Training, Tracheostomy nursing care

## Abstract

**Background:**

In many regions of the world, tracheostomy care is a major health concern. Many patients die as a result of nurses' lack of awareness regarding tracheostomy care. This study was carried out to report the acquired training, clinical experience and team approach while caring for patients with tracheostomies and then evaluate these aspects on nurses’ confidence in caring for these patients.

**Methods:**

A cross-sectional descriptive study involving a self-administered questionnaire was carried out among nurses from October 2020 to June 2021. A self-administered questionnaire including questions on sociodemographic characteristics, tracheostomy training and support and confidence in caring for patients with tracheostomies. It was disseminated electronically to the nurses working in a tertiary medical center in Saudi Arabia with multiple reminders. Group difference was determined using t test and chi square tests appropriately with a set *p* value to less than 0.05.

**Results:**

A total of 315 nurses with different educational backgrounds were included. The majority were females, middle aged and gained their nursing degree from outside the country. Over 30% of the nurses cared for patients with tracheostomies while more than two thirds did not receive adequate training to care for this patient population. Confidence was reflected in the working units, the time spent caring for these patients and the number of patients cared for. Additionally, nurses caring for children and adults with tracheostomies were more confident in their care than those caring for adolescents and older adults.

**Conclusion:**

Continuous training and competency evaluation are vital in delivering optimal care. Confidence level is impacted by training received and by the presence of assisted ventilation. Future studies should aim for a national representation of this topic to inform policy and practice.

**Supplementary Information:**

The online version contains supplementary material available at 10.1186/s12912-022-01150-3.

## Background

Tracheostomy is a surgically created opening in the trachea to allow an alternative route of respiration [1]. It is a procedure that is associated with immediate and long-term complications and requires advanced nursing care [2]. To be optimal, nursing care should be based on knowledge and skills that are built in the undergraduate programs and on-site hospital training [3]. Despite the available nursing training, knowledge levels are below optimal among nurses practicing this task with patients having tracheostomies with or without assisted ventilation.

Nurses’ knowledge and skills were evaluated before and after educational interventions in multiple studies. A study with a quasi-experimental design showed suboptimal levels of knowledge on complications of tracheostomies, tube placements and suctioning despite having a minimum of six months experience in the unit in a mix of bachelor, master and PhD holders [3]. Training on those topics improved knowledge and practice immediately after the training program and then two months after that. Another study showed that more than half of the studied nurses across two large medical centers in Saudi Arabia reported being up to date with the latest guidelines. However, their access to this information did not reflect their confidence level while caring for patients with tracheostomies [4]. On the other hand, another study in the same country showed acceptable levels of confidence among nurses caring for patients with tracheostomies although more than two thirds of the sample did not attend any competency training in the hospitals and almost half were not aware of the latest guidelines [5]. These findings were also yielded from observational studies which evaluated the nursing practices against a checklist with practice recommendations taken from guidelines. Additionally, gaps were noted between knowledge and practice among these participants [6]. Another recent study reported adequate rates of knowledge, attitude and practice in healthcare professionals including nurses while caring for critically ill patients with COVID-19. In this study, the knowledge scores were always the highest in all specialties. The correlation between the three variables, knowledge, attitude and practice were reported where higher knowledge and attitude scores were significantly correlated with higher practice scores and higher knowledge and practice scores were correlated with higher attitude scores [7]. These studies, however, lacked correlation analysis between years of experience, knowledge and confidence levels and these variables might be the cause of the conflicting findings. The role of the multidisciplinary teams with specialized nurses has been shown to impact both the nursing and the patient outcomes through reducing the length of stay, readmission rates and improving confidence levels among nurses caring for patients with tracheostomies [8]. There is continuous research about tracheostomy care but not enough literature about competencies, confidence, and knowledge about tracheostomy care practices among nurses in hospitals. Additionally, the literature lacks evidence on the impact of these variables on tracheostomy care. Therefore, the aim of this current study was to report the acquired training, clinical experience and team approach while caring for patients with tracheostomies and then evaluate these aspects on nurses’ confidence in caring for these patients.

## Methods

### Design

A cross-sectional design involving a self-administered questionnaire (Additional file 1) administered to registered nurses working in one medical center in Saudi Arabia.

### Study setting and participants

Nurses were captured from different medical and surgical wards and invited to participate in this study. Participants were included regardless of their gender, years of experience, working hours per week or their university degree. Other medical professions working in the study hospital were excluded.

### Data collection process and ethical principles

Ethical and administration approvals were granted from the hospital ethical committee and administration respectively. A link was generated on Google Forms with the link to the questionnaire of the study. Preceding the questionnaire, a paragraph was added with enough details on the objectives of the study, assumed benefit of the knowledge to be gained and the expected duration required to complete the questionnaire. Anonymity was maintained and privacy and confidentiality were assured. A sentence indicating the assumed consent from the participant with completing the questionnaire was added. The questionnaire was then disseminated to the study hospital nurses for data collection. Multiple reminders were sent following the initial invitation. Data collection took place between October 2020 and June 2021. Conserving the period to be in the acute phase of the pandemic, additional measures were taken to avoid cross-contamination and contraction of the virus. Such measures include electronic surveys and follow up with the nurses through their contact details rather than face to face dissemination of the data collection form.

### Data collection tool

The tool was developed for the current study and included questions to capture the educational background of the participants and their confidence in caring for patients with tracheostomies. The questions were yielded from previous similar studies in the literature that address similar objectives [9]. The questions addressed the perceived knowledge and confidence of the study participants therefore, no validated tool was used for this subjective assessment. Data collection was done by one trained research assistant who circulated the questionnaires and sent periodic reminders to assure a response rate. The questionnaire was divided into the following sections:

- Section one- Demographic and socio-economic data: This section included age, gender, highest nursing degree, years of experience and number of working hours per week.
- Section two- Tracheostomy training and support in the study hospital: This section addressed the population cared for, unit of work, time dedicated, and the number of patients cared for patients with tracheostomies.
- Section three- Training confidence: This section included questions on the hours of formal training acquired in the undergraduate studies and after acquiring the nursing degree, hours of clinical supervision, availability of competency training or professional development programs, and being up to date with the guidelines and advances in technology leading to the confidence in caring for patients with tracheostomies with or without assisted ventilation.

### Data analysis

Some questions of the questionnaire such as the population cared for, and the unit nurses work in were presented on a five-point Likert scale ranging between All and None. The answers were presented as such for the descriptive statistics then clustered into dichotomous categories for easier presentation of the analysis. Descriptive statistics were presented with means, standard deviation, frequencies, and percentages. Student t test was used for independent group analysis when comparing the age with other variables. Chi square test was used for comparison of categorical variables. Data was collected electronically on excel and imported into SPSS version 24 for analysis. The p value was considered significant when below 0.05.

## Results

The total number of respondents was 315 with the majority being females (*n* = 286, 90.8%) and middle-aged between 31 and 40 years (51.1%). The great majority of participants undertook their nursing bachelor’s degree (*n* = 303, 96.2%) outside Saudi Arabia (*n* = 305, 96.8%) with more than one third (34%) having 6–10 years of experience in nursing care. Most of the study participants took nursing as a full-time job with more than 40 working hours per week (*n* = 280, 88.9%). In terms of specialties, only 18.7% worked in children wards and 24.8% worked in adult units full time while the rest were floating nurses with mixed specialties that rotated based on needs. The majority worked in inpatient setting 130 (41.3%) including the intensive care unit and the operating room while the rest worked in outpatient settings such as the ENT (ear, nose throat) unit and the clinics. The details of the participant characteristics are presented in Table 1.

**Table 1:** Presentation of the study characteristics, training, and confidence among the study participants (*N* = 315, 100%)

Variables	**Categories**	**Total** **(315, 100%)**
Age*		35.71 (7.05)
gender	Male	29 (9.2)
	Female	286 (90.8)
Degree location	Saudi Arabia	10 (3.2)
	Outside Saudi Arabia	304 (96.5)
Nursing degree	Bachelor	302 (95.9)
	Master	12 (3.8)
Years of experience	1–5	41 (13)
	6–10	110 (34.9)
	11–15	79 (25.1)
	more than 15	85 (27)
Hours/week	1–9	8 (2.5)
	10–19	10 (3.2)
	20–29	5 (1.6)
	30–39	12 (3.8)
	more than 40	280 (88.9)
Patient population		
Children (0–11)	None	171 (54.3)
	Some	38 (12.1)
	Half	11 (3.5)
	Most	36 (11.4)
	All	59 (18.7)
Adolescent (12–17)	None	138 (43.8)
	Some	88 (27.9)
	Half	26 (8.3)
	Most	30 (9.5)
	All	33 (10.5)
Adults (18–65)	None	79 (25.1)
	Some	49 (15.6)
	Half	18 (5.7)
	Most	91 (28.9)
	All	78 (24.8)
Old (> 65)	None	118 (37.5)
	Some	42 (13.3)
	Half	23 (7.3)
	Most	70 (22.2)
	All	62 (19.7)
Working department		
Emergency Department	None	224 (71.1)
	Some	20 (6.3)
	Half	8 (2.5)
	Most	14 (4.4)
	All	49 (15.6)
Intensive Care Unit	None	220 (69.8)
	Some	22 (7)
	Half	13 (4.1)
	Most	18 (5.7)
	All	42 (13.3)
Operative Room	None	282 (89.5)
	Some	20 (6.3)
	Half	5 (1.6)
	Most	1 (0.3)
	All	7 (2.2)
Inpatient	None	79 (25.1)
	Some	48 (15.2)
	Half	15 (4.8)
	Most	43 (13.7)
	All	130 (41.3)
Outpatient	None	282 (89.5)
	Some	16 (5.1)
	Half	12 (3.8)
	All	5 (1.6)
ENT Service	None	280 (88.9)
	Some	14 (4.4)
	Half	11 (3.5)
	Most	3 (1)
	All	7 (2.2)
Other	None	281 (89.2)
	Some	17 (5.4)
	Half	7 (2.2)
	Most	4 (1.3)
	All	6 (1.9)

^a^Presented in means and standard deviations

### Study outcomes

Although almost one third of the study participants (30.2%) spent more than 50% of their time caring for patients with tracheostomies, only 18.7% cared for more than 50 patients with this condition. Surprisingly, 15.9% of the participants did not receive any formal training prior to caring for these patients while almost half (48.6%) received minimal training of 1–5 h. Similarly, following graduation 18.4% had no clinical supervision prior to caring for these patients while 76.8% reported having a formal tracheostomy competency training program in their departments.

In terms of updates, almost two thirds (65.4%) felt they were up to date with evidence-based practice while less than half (46.3%) were up to date with the technology advances related to tracheostomies. When asked about the type of training they believed to benefit them in managing this patient population, more than half (56.8%) preferred postgraduate workshops. The great majority (77.1%) reported having expert clinical support within their multidisciplinary team for caring for patients with tracheostomies while this percentage dropped to 39.7% when the patients were on assisted ventilation.

In terms of confidence, slightly more than half (52.4%) reported feeling confident to manage patients with tracheostomies while this percentage dropped again (34.6%) when the patients were on assisted ventilation. These details of the clinical training, updates and confidence are presented in Table 2.

**Table 2:** Presentation of clinical training, updates and confidence among the study participants (*N* = 315)

Variables	Categories	Total (315, 100%)
Within the last year what percentage of your active clinical time consisted of the management of patients with a tracheostomy?	None	11 (3.5)
	1–9%	42 (13.3)
	10–24%	85 (27)
	25–49%	82 (26)
	50% or more	95 (30.2)
Please indicate how many patients you have worked with who are tracheotomized and ventilator assisted?	None	39 (12.4)
	1–10	130 (41.3)
	11–15	30 (9.5)
	16–50	57 (18.1)
	more than 50	59 (18.7)
Prior to treating patients independently, approximately how many hours formal training did you receive in tracheostomy management?	None	50 (15.9)
	1–5	153 (48.6)
	6–10	54 (17.1)
	11–20	20 (6.3)
	more than 20	38 (12.1)
Prior to treating patients independently, how many hours of clinical supervision did you gain in tracheostomy management (postgraduate)?	None	58 (18.4)
	1–5	139 (44.1)
	6–10	54 (17.1)
	11–20	19 (6)
	more than 20	45 (14.3)
Does your department have a formal tracheostomy competency training program?	Yes	242 (76.8)
Updates and confidence		
Do you feel up to date with the available evidence-based practice in tracheostomy management?	No	95 (30.2)
	Yes	206 (65.4)
	Not sure	14 (4.4)
Do you feel up to date with the advances in tracheostomy technology including the specialized tracheostomy tube options available?	No	160 (50.8)
	Yes	146 (46.3)
	Sometimes	9 (2.9)
What training, if any, would you find beneficial to assist you in managing patients who are tracheostomized?	No training	47 (14.9)
	Internship rotation in ENT services	37 (11.7)
	Undergraduate courses	52 (16.5)
	Postgraduate workshops	179 (56.8)
Do you feel you have expert clinical support within your multidisciplinary team for the management of patients with a tracheostomy and those patients requiring ventilator assistance? [Tracheostomy only]	Yes	243 (77.1)
Do you feel you have expert clinical support within your multidisciplinary team for the management of patients with a tracheostomy and those patients requiring ventilator assistance? [Tracheostomy + ventilator-assistance]	Yes	125 (39.7)
Does the setting in which you work have an optimal team approach for the management of patients with a tracheostomy?	No	58 (18.4)
	Yes	243 (77.1)
	Sometimes	14 (4.4)
Do you feel confident to manage the majority of patients with a tracheostomy within your team	Very confident	44 (14)
	Confident	121 (38.4)
	Neutral	112 (35.6)
	Not very confident	31 (9.8)
	Not at all confident	7 (2.2)
Do you feel confident to manage the patients with a tracheostomy who also require ventilator assistance?	Very confident	28 (8.9)
	Confident	81 (25.7)
	Neutral	108 (34.3)
	Not very confident	54 (17)
	Not all confident	44 (14)

### Main results

When evaluating the group differences, it was noted that there were more females than males working in the emergency department (*n* = 77, 84.6% vs. *n* = 14, 15.4%; *p* = 0.016) and ICU (Intensive care unit) (*n* = 80, 84.2% vs *n* = 15, 15.8%, *p* = 0.008) while no other gender differences were noted in other departments. In terms of confidence, those who worked all the time with children and adults where significantly more confident in working with the respective patient population even with assisted ventilation than their counterparts. In contrast, those working with older adults and adolescents lacked confidence in caring for patients with tracheostomies but those who worked with older adults showed significant confidence when the patient required assisted ventilation. The details of the patient population categories against the confidence are presented in Table 3. Similarly, those who worked in the emergency department, ICU, and inpatient departments had more confidence in their management of patients with tracheostomies when compared to nurses of other units while emergency department nurses lost this confidence when the patients were also on assisted ventilation. These details are presented in Table 4. Further on confidence, it was noted that those who spend more time caring for patients with tracheostomies or provided care for more of these patients, had more formal training hours, better clinical supervision and a competency training program in their units were more confident to work with patients having tracheostomies and on assisted ventilation than their counterparts. This is true for all except for those who spent more than 50% of their time caring for these patients which lost its significance when assisted ventilation was introduced as presented in Table 5. Similarly, those who belief they are up to date with evidence-based practice and the latest technologies were significantly more confident when caring for patients with tracheostomies with and without assisted ventilation. While confidence also reflected their belief in their clinical team as presented in Table 6.

**Table 3:** Presentation of the patient population and the confidence among the study participants (*N* = 315, 100%)

**Variables**	**Category**	**Confidence in Managing patients with tracheostomies**		**Total**	***P*-value**
		**Confident** **(n, %)**	**Not confident** **(*n* = , %)**		
Children (0–11)	No	108 (53.2%)	14 (6.9%)	122 (60.1%)	0.001
	Yes	57 (28.1%)	24 (11.8%)	81 (39.9%)	
Adolescent (12–17)	No	73 (36%)	18 (8.9%)	91 (44.8%)	0.727
	Yes	92 (45.3%)	20 (9.9%)	112 (55.2%)	
Adults (18–65)	No	27 (13.3%)	13 (6.4%)	40 (19.7%)	0.013
	Yes	138 (68%)	25 (12.3%)	163 (80.3%)	
Older adults (> 65)	No	50 (24.6%)	15 (7.4%)	65 (32%)	0.275
	Yes	115 (56.7%)	23 (11.3%)	138 (68%)	
		**Confidence in managing patients with tracheostomies on Ventilators**		**Total**	***P*-value**
		**Confident** **(n, %)**	**Not confident** **(*n* = , %)**		
Children (0–11)	No	72 (34.8%)	46 (22.2%)	118 (57%)	0.006
	Yes	37 (17.9%)	52 (25.1%)	89 (43%)	
Adolescents (12–17)	No	43 (20.8%)	51 (24.6%)	94 (45.4%)	0.069
	Yes	66 (31.9%)	47 (22.7%)	113 (54.6%)	
Adults (18–65)	No	15 (7.2%)	38 (18.4%)	53 (25.6%)	0.000
	Yes	94 (45.4%)	60 (29%)	154 (74.4%)	
Older adults (> 65)	No	29 (14%)	45 (21.7%)	74 (35.7%)	0.016
	Yes	80 (38.6%)	53 (25.6%)	133 (64.3%)

**Table 4:** Presentation of departments and confidence among the study population (*N* = 315, 100%)

**Variables**	**Category**	**Confidence in Managing patients with tracheostomies**		**Total**	***P*-value**
		**Confident** **(n, %)**	**Not confident** **(n, %)**		
Emergency Department	No	132 (65%)	21 (10.3%)	153 (75.4%)	0.001
	Yes	33 (16.3%)	17 (8.4%)	50 (24.6%)	
Intensive Care Unit	No	103 (50.7%)	33 (16.3%)	136 (67%)	0.004
	Yes	62 (30.5%)	5 (2.5%)	67 (33%)	
Operative Room	No	149 (73.4%)	35 (17.2%)	184 (90.6%)	0.731
	Yes	16 (7.9%)	3 (1.5%)	19 (9.4%)	
Inpatient	No	38 (18.7%)	16 (7.9%)	54 (26.6%)	0.016
	Yes	127 (62.6%)	22 (10.8%)	149 (73.4%)	
Outpatient	No	156 (76.8%)	33 (16.3%)	189 (93.1%)	0.091
	Yes	9 (4.4%)	5 (2.4%)	14 (6.9%)	
ENT Service	No	154 (75.9%)	34 (16.7%)	188 (92.6%)	0.412
	Yes	11 (5.4%)	4 (2%)	15 (7.4%)	
Other	No	149 (73.4%)	34 (16.4%)	183 (90.1%)	0.877
	Yes	16 (7.9%)	4 (2%)	20 (9.9%)	
		**Confidence in Managing patients with tracheostomies on Ventilators**		**Total**	***P*-value**
		**Confident** **(n, %)**	**Not confident** **(n, %)**		
Emergency Department	No	79 (38.2%)	79 (38.2%)	158 (76.3%)	0.169
	Yes	30 (14.5%)	19 (9.2%)	49 (23.7%)	
Intensive Care Unit	No	54 (26.1%)	93 (44.9%)	147 (71%)	0.000
	Yes	55 (26.6%)	5 (2.4%)	60 (29%	
Operative Room	No	94 (45.4%)	92 (44.4%)	186 (89.9%)	0.069
	Yes	15 (7.2%)	6 (2.9%)	21 (10.1%)	
Inpatient	No	38 (18.4%)	14 (6.8%)	52 (25.1%)	0.001
	Yes	71 (34.4%)	84 (40.6%)	155 (74.9%)	
Outpatient	No	100 (48.3%)	90 (43.5%)	190 (91.8%)	0.980
	Yes	9 (4.3%)	8 (3.9%)	17 (8.2%)	
ENT Service	No	100 (48.3%)	91 (44%)	191 (92.3%)	0.764
	Yes	9 (4.3%)	7 (3.4%)	16 (7.7%)	
Other	No	97 (46.9%)	90 (43.5%)	187 (90.3%)	0.489
	Yes	12 (5.8%)	8 (3.9%)	20 (9.7%)

**Table 5:** Presentation of training and supervision with confidence among the study participants (*N* = 315, 100%)

**Variables**		**Confidence in Managing patients with tracheostomies**		**Total**	***P*-value**
		**Confident** **(n, %)**	**Not confident (n, %)**		
Within the last year what percentage of your active clinical time consisted of the management of patients with a tracheostomy?	None	2 (1.2%)	6 (15.8%)	8 (3.9%)	0.000
	1–9%	24 (14.5%)	3 (7.9%)	27 (13.3%)	
	10–24%	38 (23%)	16 (42.1%)	54 (26.6%)	
	25–49%	34 (20.6%)	7 (18.4%)	41 (20.2%)	
	50% or more	67 (40.7%)	6 (15.8%)	73 (36%)	
Please indicate how many patients you have worked with who are tracheotomized and ventilator assisted?	None	15 (6.1%)	8 (21.1%)	23 (11.3%)	0.041
	1–10	60 (36.4%)	18 (47.4%)	78 (38.4%)	
	11–15	20 (12.1%)	5 (13.2%)	25 (12.3%)	
	16–50	29 (17.6%)	4 (12.1%)	33 (16.3%)	
	more than 50	41 (24.8%)	3 (7.9%)	44 (21.7%)	
Prior to treating patients independently, approximately how many hours formal training did you receive in tracheostomy management?	None	11 (6.7%)	12 (31.6%)	23 (11.3%)	0.000
	1–5	83 (50.3%)	21 (55.3%)	104 (51.2%)	
	6–10	27 (16.4%)	3 (7.9%)	30 (14.8%)	
	11–20	12 (7.3%)	2 (5.3%)	14 (6.9%)	
	more than 20	32 (15.8%)	0 (0%)	32 (15.8%)	
Prior to treating patients independently, how many hours of clinical supervision did you gain in tracheostomy management (postgraduate)?	None	14 (8.5%)	17 (44.7%)	31 (15.5%)	0.000
	1–5	80 (48.5%)	13 (34.2%)	93 (45.8%)	
	6–10	25 (15.2%)	5 (13.2%)	30 (14.8%)	
	11–20	11 (6.7%)	2 (5.3%)	13 (6.4%)	
	more than 20	35 (21.2%)	1 (2.6%)	36 (17.7%)	
Does your department have a formal tracheostomy competency training program?		148 (89.7%)	21 (55.3%)	169 (83.3%)	0.000
		**Confidence in managing patients with tracheostomies on Ventilators**		**Total**	***P*-value**
		**Confident** **(n, %)**	**Not confident (n, %)**		
Within the last year what percentage of your active clinical time consisted of the management of patients with a tracheostomy?	None	2 (1.8%)	6 (6.1%)	8 (3.9%)	0.108
	1–9%	15 (13.8%)	10 (10.2%)	25 (12.1%)	
	10–24%	25 (22.9%)	30 (30.6%)	55 (26.6%)	
	25–49%	22 (20.2%)	25 (25.5%)	47 (22.7%)	
	50% or more	45 (41.3%)	27 (27.6%)	72 (34.8%)	
Please indicate how many patients you have worked with who are tracheotomized and ventilator assisted?	None	4 (3.7%)	19 (82.6%)	23 (11.1%)	0.001
	1–10	44 (40.4%)	41 (41.8%)	85 (41.1%)	
	11–15	9 (8.3%)	12 (12.2%)	21 (10.1%)	
	11–50	20 (18.3%)	14 (14.3%)	34 (16.4%)	
	more than 50	32 (29.4%)	12 (12.2%)	44 (21.3%)	
Prior to treating patients independently, approximately how many hours formal training did you receive in tracheostomy management?	None	7 (6.4%)	20 (20.4%)	27 (13%)	0.000
	1–5	50 (45.9%)	58 (59.2%)	108 (52.2%)	
	6–10	20 (18.3%)	8 (8.2%)	28 (13.5%)	
	11–20	10 (9.2%)	5 (5.1%)	15 (7.2%)	
	more than 20	22 (20.2%)	7 (7.1%)	29 (14%)	
Prior to treating patients independently, how many hours of clinical supervision did you gain in tracheostomy management (postgraduate)?	None	9 (8.3%)	30 (30.6%)	39 (18.8%)	0.000
	1–5	50 (45.9%)	42 (42.9%)	92 (44.4%)	
	6–10	16 (14.7%)	15 (15.3%)	31 (15%)	
	11–20	6 (5.5%)	6 (6.1%)	12 (5.8%)	
	more than 20	28 (25.7%)	5 (5.1%)	33 (15.9%)	
Does your department have a formal tracheostomy competency training program?		99 (90.8%)	74 (75.5%)	173 (83.6%)	0.003

**Table 6:** Presentation of updates and clinical support with confidence among the study participants (*N* = 315)

**Variables**		**Confidence in Managing patients with tracheostomies**		**Total**	***P*-value**
		**Confident** **(n, %)**	**Not confident (n, %)**		
Do you feel up to date with the available evidence-based practice in tracheostomy management?	No	21 (12.7%)	15 (39.5%)	36 (17.7%)	0.000
	Yes	141 (85.5%)	17 (44.7%)	158 (77.8%)	
	Total	165 (100%)	38 (100%)	203 (100%)	
Do you feel up to date with the advances in tracheostomy technology including the specialized tracheostomy tube options available?	No	53 (32.1%)	28 (73.3%)	81 (39.9%)	0.000
	Yes	108 (65.5%)	8 (21.1%)	116 (57.1%)	
	Total	165 (100%)	38 (100%)	203 (100%)	
What training, if any, would you find beneficial to assist you in managing patients who are tracheostomized?	No training	16 (9.7%)	12 (31.6%)	28 (13.8%)	0.002
	Internship rotation in ENT services	18 (10.9%)	3 (7.9%)	21 (10.3%)	
	Undergraduate courses	25 (15.2%)	8 (21.1%)	33 (16.3%)	
	Postgraduate workshops	106 (64.2%)	15 (39.5%)	121 (59.6%)	
	Total	165 (100%)	38 (100%)	203 (100%)	
Do you feel you have expert clinical support within your multidisciplinary team for the management of patients with a tracheostomy and those patients requiring ventilator assistance? [Tracheostomy only]	No	24 (14.5%)	17 (44.7%)	41 (20.2%)	0.000
	Yes	141 (85.5%)	21 (55.3%)	162 (79.8%)	
	Total	165 (100%)	38 (100%)	203 (100%)	
Do you feel you have expert clinical support within your multidisciplinary team for the management of patients with a tracheostomy and those patients requiring ventilator assistance? [Tracheostomy + ventilator-assistance]	No	86 (52.1%)	29 (76.3%)	115 (56.7%)	0.007
	Yes	79 (47.9%)	9 (23.7%)	88 (43.3%)	
	Total	165 (100%)	38 (100%)	203 (100%)	
Does the setting in which you work have an optimal team approach for the management of patients with a tracheostomy?	No	13 (7.9%)	19 (50%)	32 (15.8%)	0.000
	Yes	150 (90.9%)	14 (36.8%)	164 (80.8%)	
	Total	165 (100%)	38 (100%)	203 (100%)	
		**Confidence in Managing patients with tracheostomies on Ventilators**		**Total**	***P*-value**
		**Confident** **(n, %)**	**Not confident (n, %)**		
Do you feel up to date with the available evidence-based practice in tracheostomy management?	No	14 (12.8%)	42 (42.9%)	56 (27.1%)	0.000
	Yes	92 (84.4%)	48 (49%)	140 (67.6%)	
	Total	109 (100%)	98 (100%)	207 (100%)	
Do you feel up to date with the advances in tracheostomy technology including the specialized tracheostomy tube options available?	No	37 (33.9%)	67 (68.4%)	104 (50.2%)	0.000
	Yes	69 (63.3%)	30 (30.6%)	99 (47.8%)	
	Total	109 (100%)	98 (100%)	207 (100%)	
What training, if any, would you find beneficial to assist you in managing patients who are tracheostomized?	No training	11 (10.1%)	23 (23.5%)	34 (16.4%)	0.057
	Internship rotation in ENT services	11 (10.1%)	9 (9.2%)	20 (9.7%)	
	Undergraduate courses	18 (16.5%)	10 (10.2%)	28 (13.5%)	
	Postgraduate workshops	69 (63.3%)	56 (57.1%)	125 (60.4%)	
	Total	109 (100%)	98 (100%)	207 (100%)	
Do you feel you have expert clinical support within your multidisciplinary team for the management of patients with a tracheostomy and those patients requiring ventilator assistance? [Tracheostomy only]	No	20 (18.3%)	23 (23.5%)	43 (20.8%)	0.365
	Yes	89 (81.7%)	75 (76.5%)	164 (79.2%)	
	Total	109 (100%)	98 (100%)	207 (100%)	
Do you feel you have expert clinical support within your multidisciplinary team for the management of patients with a tracheostomy and those patients requiring ventilator assistance? [Tracheostomy + ventilator-assistance]	No	39 (35.8%)	88 (89.8%)	127 (61.4%)	0.000
	Yes	70 (64.2%)	10 (10.2%)	80 (38.6%)	
	Total	109 (100%)	98 (100%)	207 (100%)	
Does the setting in which you work have an optimal team approach for the management of patients with a tracheostomy?	No	27 (27.6%)	8 (7.3%)	35 (16.9%)	0.000
	Yes	100 (91.7%)	64 (65.3%)	164 (79.2%)	
	Total	109 (100%)	98 (100%)	207 (100%)	

## Discussion

The aim of this study was to report the training hours before and after graduation and the clinical experience of nurses caring for patients with tracheostomies with and without assisted ventilation. The other aim was to evaluate the effect of these aspects on the confidence level among the practicing nurses. This was done through a cross-sectional approach of nurses working across in and out-patient units in one medical facility in Saudi Arabia. The main results showed that almost half the sample did not receive adequate training prior to caring for these patients which might explain the suboptimal level of knowledge and care reported by Abdelazeem et al. [3] in a similar study setting. The majority thought that postgraduate workshops to be the optimal platform for delivering the updated guidelines and improving tasks which was also reported to be the preferred platform in other studies [10]. In addition to being the preferred methods of education by the end-users, nurses, it was also shown that hands on training by a specialized tracheostomy team improved methods of insertion and care of patients with tracheostomies [11] and their confidence in their management. To support that, Sodhi et al. [12] demonstrated a decrease in the number of ICU readmissions after the formation of the tracheostomy care nurse program where tracheostomy care nurses were better in managing emergency tube changes and hypoxia.

Confidence was a significant construct in this study, which was impacted by the patient population and the working department. It was seen that those who worked with children having tracheostomies were more confident in caring for this population than those who worked with adolescents or older adults. This may be because nurses working in these units are more exposed to tracheostomies than nurses caring for other patient populations as reported by Deutsch [13]. This is due to that fact that children are more tolerable than adults to extended intubation making these procedures more often performed in children than in adults [14]. Additionally, those who worked in the emergency department, ICU, and inpatient departments had more confidence in their management of patients with tracheostomies when compared to nurses of other units while emergency department nurses lost this confidence when the patients were also on assisted ventilation. This is because nurses in emergency departments are less likely to deal with ventilated patients who require a long hospital stay [15]. Along the same line, confidence was impacted by the clinical time spent caring for these patients, previous training, clinical supervision and the availability of competency training programs in the working departments. Confidence was also a construct of interest in previous studies which reported higher levels with departmental training programs [4], formal training and clinical supervision [14].

Despite all the previously reported findings, clinical training hours and clinical supervision were not completely performed in this current and other similar studies [4]. On the other hand, the positive impact of a multidisciplinary team approach was highlighted in caring for patients having tracheostomies in this current study as well as in previous studies which confirmed that this team approach improved outcomes of patients in the COVID-19 era and improved the confidence levels of the nurses [16]. Considering the demands on the hospitals and the increasing rates of tracheostomies and assisted ventilation during the COVID-19 pandemic, McGrath et al. [17] highlighted the importance of guidance and education for the nurses working outside the intensive care units as their services may be needed when the ICUs are overwhelmed. This might be proven correct in the current study setting were these nurses received minimal training and lacked confidence in managing patients with tracheostomies.

Consequently, this study recommends delivering more tracheostomies care practical sessions to nurses in different work settings and giving them remarkable knowledge that will increase their confidence in tracheostomy management. Also, adjusting the nursing program to have vivid and precise undergraduate training and simulation for tracheostomy care in all nursing institutions. It is also important for nurses to learn from professional and experienced coworkers and to show personal educational effort by staying up to date with evidence-based tracheostomy related advancements. Certain studies have recommended adding tracheostomy care into basic life support courses (BLS) to ensure airway patency [18].

In terms of limitations, the used tool and the lack of generalizability raise some concern. Future studies should be conducted with tools addressing the care of patients having tracheostomy more accurately. This means that questions addressing the care delivered in general to patients with tracheostomy or the time dedicated for the care of the tracheostomy itself. This information will be predictive of the time spent with patients having this condition and is more likely to inform practice. Although the sample size in this study is a strength, generalizability remains lacking as this study was conducted in one site only and the majority of the sample was female nurses. The latter draws major concerns in terms of generalizing the findings and informing practice. Additionally, the different educational background of the study participants reflected in the large number of participants having their education from outside the country confounds the study outcome and limits the possibility of drawing clear conclusions about the study variables. The design of the study does not allow for direct observation of the participants performance and therefore, the accuracy of the findings in terms of real practice performance is of concern. Considering the conflicting results acquired from other studies in the same country, a multi-site/national study is recommended to allow for a better understanding of the educational needs of the nurses and the confidence gained through different aspects.

## Conclusion

Clinical training before and after graduation have a positive impact on the care provided for patients with tracheostomies. A considerable number reported caring for patients with tracheostomy and assisted ventilation, a relatively small number reported being trained for this type of care or being up to date with the guidelines. This reflected the nurses’ confidence in delivering this type of care which was greatly impacted when assisted ventilation was added. Therefore, continuous training and competency evaluation are vital in delivering optimal care. Future studies should aim for a national representation of nurses working with this patient population to gain a better understanding of their educational and training needs and inform policy and practice.

## Supplementary Information

**Additional file 1.** Questionnaire.

## Data Availability

The data will be available with he corresponding author as and when requested.

## Abbreviations

BLS: Basic Life Support Courses
COVID-19: Corona Virus Disease- 2019
ICU: Intensive Care Unit
ENT: Ear Nose Throat

## References

[1] Abdulrahman E, Musa MT, Eltayeb RM, Ali Fadlalmola H (2021). Effect of an Educational Training Program in Tracheostomy Care on Nurses' Knowledge and Skills. Int J Nurs Educ.

[2] Cheung NH, Napolitano LM (2014). Tracheostomy: Epidemiology, Indications, Timing, Technique, and OutcomesDiscussion. Respir Care.

[3] Abdelazeem E, Fashafsheh I, Fadllalah H (2019). Effect of training program on nurses knowledge and competence regarding endotracheal tube and tracheostomy care in mechanically ventilated patients. International J Nurs.

[4] Alja'afreh MA, Mosleh SM, Habashneh SS. Nurses’ perception and attitudes towards oral care practices for mechanically ventilated patients. Saudi Med J. 2018;39:379–85. 10.15537/smj.2018.4.21749 PMC5938652 29619490

[5] Alnemare AK (2020). Nurses training and confidence in management of tracheostomy patients in a community hospital in Saudi Arabia. J Res Med Dental Sci.

[6] Day T, Farnell S, Haynes S, Wainwright S, Wilson-Barnett J (2002). Tracheal suctioning: an exploration of nurses' knowledge and competence in acute and high dependency ward areas. J Adv Nurs.

[7] Nepal R, Sapkota K, Adhikari K, Paudel P, Adhikari B, Paudyal N (2020). Knowledge, attitude and practice regarding COVID-19 among healthcare workers in Chitwan, Nepal.

[8] Bonvento B, Wallace S, Lynch J, Coe B, McGrath BA (2017). Role of the multidisciplinary team in the care of the tracheostomy patient. J Multidiscip Healthc.

[9] Alnemare (2020). Nurses training and confidence in management of tracheostomy patients in a community hospital in Saudi Arabia. J Res Med Dental Sci.

[10] Khanum T, Zia S, Khan T, Kamal S, Khoso MN, Alvi J, Ali A. Assessment of knowledge regarding tracheostomy care and management of early complications among healthcare professionals. Braz J Otorhinolaryngol. 2022;88:251–6. 10.1016/j.bjorl.2021.06.011 PMC9422647 34419386

[11] Twose P, Jones G, Lowes J, Morgan P (2019). Enhancing care of patients requiring a tracheostomy: a sustained quality improvement project. J Crit Care.

[12] Sodhi K, Shrivastava A, Singla MK (2014). Implications of dedicated tracheostomy care nurse program on outcomes. J Anesth.

[13] Deutsch ES (2010). Tracheostomy: pediatric considerations. Respir Care.

[14] Nagler J, Auerbach M, Monuteaux MC, Cheek JA, Babl FE, Oakley E, Nguyen L, Rao A, Dalton S, Lyttle MD (2021). Exposure and confidence across critical airway procedures in pediatric emergency medicine: An international survey study. Am J Emerg Med.

[15] Rose L, Gray S, Burns K, Atzema C, Kiss A, Worster A, Scales DC, Rubenfeld G, Lee J (2012). Emergency department length of stay for patients requiring mechanical ventilation: a prospective observational study. Scand J Trauma Resusc Emerg Med.

[16] Ali A, Kane D, Mangahas T, Kane L, Yavarovich E, Lamb C (2021). Tracheostomy training and troubleshooting: a pandemic educational effort. Chest.

[17] McGrath B, Ashby N, Birchall M, Dean P, Doherty C, Ferguson K, Gimblett J, Grocott M, Jacob T, Kerawala C (2020). Multidisciplinary guidance for safe tracheostomy care during the COVID-19 pandemic: the NHS National Patient Safety Improvement Programme (NatPatSIP). Anaesthesia.

[18] Parker V, Giles M, Shylan G, Austin N, Smith K, Morison J, Archer W (2010). Tracheostomy management in acute care facilities–a matter of teamwork. J Clin Nurs.

