# Supplementary material for: Attitude and practices of tracheostomy care among nursing staff in Saudi Arabia
Source: BMC Nurs. 2022 Dec 23;21:367. doi: 10.1186/s12912-022-01150-3 (PMC9780093; doi:10.1186/s12912-022-01150-3)
Supplement: Supplementary file 1 — Additional file 1. Questionnaire. [file 12912_2022_1150_MOESM1_ESM.docx]

**Dear Nurses , we are conducting a research to evaluate tracheostomy care among Nurses in KFMC . If you agreed on participation , kindly answer all questions**

**1- Agree 2- Don’t agree**

- ***Section one : Demographic & socio-economic Data :***

**1- Where did you undertake your degree in Nursing ?**

**1- Saudi Arabia 2- others – please specify :**

**2- From which university did you receive your degree in Nursing ? ( please write )**

- **Bachelors :**
- **Masters :**
- **PhD**

**4- How many years have you been practicing Nursing Care ?**

1. **1-5 3- 11-15**
2. **6-10 4- more than 15**

**5- How may paid hours per week do you work as Nurse ?**

1. **1-9 4- 30-39**
2. **10-19 5- 40 +**
3. **20-29**

- ***Section two : Tracheostomy training and support in KAUH .***

**6- What proportion of your active clinical time is spent working with the following populations :**

**(NB this does not include time dedicated to administration tasks such as teaching, research, etc)**

**All Most Half Some None**

**Children (0–11yrs) 1 2 3 4 5**

**Adolescents (12–17yrs) 1 2 3 4 5**

**Adults (18–65yrs) 1 2 3 4 5**

**Seniors (65+ years) 1 2 3 4 5**

**7- In which of the listed job setting(s) do you currently manage patients with tracheostomies?**

**( If you work in more than one setting, please estimate the percentage of time you currently work in each )**

**All Most Half Some None**

**Emergency Department 1 2 3 4 5**

**Intensive Care Unit 1 2 3 4 5**

**Operating Room 1 2 3 4 5**

**Inpatient 1 2 3 4 5**

**Outpatient 1 2 3 4 5**

**ENT service 1 2 3 4 5**

**Others 1 2 3 4 5**

**8- Within the last year what percentage of your  active clinical time consisted of the management of patients with a tracheostomy?**

1. **None 4- 25-49 %**
2. **1-9 % 5- 50 %**
3. **10-24 %**

**9- Please indicate how many patients you have worked with who are tracheotomised and ventilator assisted ?**

1. **None 3- 11-5**
2. **1-10 4- more than 50**

- ***Section three : Training confidence .***

**10- Prior to treating patients independently, approximately how many hours formal training (i.e., undergraduate lectures, workshops) did you receive in tracheostomy management?**

1. **None 3- 11–20**
2. **1–5 4- More than 20**

**11- Prior to treating patients independently, how many hours of clinical supervision did you gain in tracheostomy management ( postgraduate )?**

1. **None 3- 11–20**
2. **1–5 4- More than 20**

**12 - Does your department have a formal tracheostomy competency training program?**

1. **Yes 2- Currently developing one 3- No**

**13- Please indicate what tracheostomy-related professional develop- ment activities you have undertaken? ( you can choose more than one )**

1. **Workshops**
2. **Conference**
3. **Visited specialist center(s)**
4. **Teleconferences**
5. **Becoming a member of an interest group( e.g.,Tracheostomy Interest Group)**

- **Other ________________________________**

**14- Do you feel up to date with the available evidence-based practice in tracheostomy management?**

1. **Yes 2- Not sure 3-No**

**Comments:_____________________________________________________**

**15- Do you feel up to date with the advances in tracheostomy technology including the specialized tracheostomy tube options available ?**

**(e.g., ‘talking’ traches, double cuffed tubes, foam cuffs, metal tubes, extra long cuffs)**

1. **Yes 2- Sometimes 3- No**

**16-What training, if any, would you find beneficial to assist you in managing patients who are tracheostomized?**

1. **No training**
2. **Undergraduate courses**
3. **Postgraduate workshops**
4. **Internship rotation in ENT services.**

**17- Do you feel you have expert clinical support within your multidisciplinary team for the management of patients with a tracheostomy and those patients requiring ventilator assistance?**

1. **Tracheostomy only. 1- Yes 2- No**
2. **Tracheostomy and requiring ventilator assistance 1- Yes 2- No**

**18- Does the setting in which you work have an optimal team approach for the management of patients with a tracheostomy?**

**1- Yes 2- Sometimes 3-No**

**Comments:_________________________________________________________**

**19- Do you feel confident to manage the majority of patients with a tracheostomy within your team?**

1. **Not at all confident 4- Confident**
2. **Not very confident 5-- Very confident**
3. **Neutral**

**Comments:_________________________________________________________**

**20- Within your multidisciplinary team, do you feel confident in managing patients with a tracheostomy who also require ventilator assistance?**

1. **Not at all confident 4- Confident**
2. **Not very confident 5- Very confident**
3. **Neutral**

**Comments:________________________________________________**

**Thank you**

Together we will be better
